# *Drosophila* PEBP1 inhibits intestinal stem cell aging via suppression of ERK pathway

**DOI:** 10.18632/oncotarget.24834

**Published:** 2018-04-06

**Authors:** Jung-Hoon Pyo, Ho-Jun Jeon, Joung-Sun Park, Jae-Sun Lee, Hae-Young Chung, Mi-Ae Yoo

**Affiliations:** ^1^ Department of Molecular Biology, Pusan National University, Busan, Republic of Korea; ^2^ Institute of Systems Biology (ISB), Pusan National University, Busan, Republic of Korea; ^3^ Molecular Inflammation Research Center for Aging Intervention (MRCA), College of Pharmacy, Pusan National University, Busan, Republic of Korea; ^4^ Department of Molecular Medicine and Hypoxia-Related Disease Research Center, Inha University College of Medicine, Incheon, Republic of Korea

**Keywords:** Drosophila, intestinal stem cell, stem cell aging, PEBP1, ERK suppressor, Gerotarget

## Abstract

The intestine is a high cellular turnover tissue largely dependent on the regenerative function of stem cell throughout life, and a signaling center for the health and viability of organisms. Therefore, better understanding of the mechanisms underlying the regulation of intestinal stem cell (ISC) regenerative potential is essential for the possible intervention of aging process and age-related diseases. *Drosophila* midgut is a well-established model system for studying the mechanisms underlying ISC regenerative potential during aging. Here, we report the requirement of *Drosophila* phosphatidylethanolamine binding protein 1 (PEBP1) in ISC regenerative potential. We showed that PEBP1 was strongly expressed in enterocytes (ECs) of guts and its decrease with age and oxidative stress. Furthermore, the downregulation of PEBP1 in ECs accelerates ISC aging, as evidenced by ISC hyper-proliferation, γH2AX accumulation, and centrosome amplification, and intestinal hyperplasia. The decrease in PEBP1 expression was associated with increased extracellular signal-regulated kinase (ERK) activity in ECs. All these phenotypes by EC-specific depletion of PEBP1 were rescued by the concomitant inhibition of ERK signaling. Our findings evidence that the age-related downregulation of PEBP1 in ECs is a novel cause accelerating ISC aging and that PEBP1 is an EC-intrinsic suppressor of epidermal growth factor receptor (EGFR)/ERK signaling. Our study provides molecular insights into the tight regulation of EGFR/ERK signaling in niches for stem cell regenerative potential.

## INTRODUCTION

The intestine is not only essential for the uptake of nutrients but also acts as an effective barrier and protects organs from toxins and pathogens in the intestinal lumen. Furthermore, the intestine is a signaling center for the health and viability of the organisms [1]. As the intestinal epithelium loses millions of cells daily during tissue functions and by environmental exposure, the intestine imposes a requirement for astounding renewal capacity throughout life, which is driven by the resident intestinal stem cells (ISCs) that reside within a specialized niche [2–5]. Thus, better understanding of the mechanisms underlying the maintenance of ISC regenerative potential is essential for the possible intervention of aging process and age-related diseases.

The midgut of *Drosophila* is a well-established model system for studying ISC regenerative potential [6–9]. *Drosophila* ISC are multipotent and give rise to both enterocytes (ECs) and enteroendocrine cells (EEs) either directly or through an intermediate state of enteroblasts (EBs) [7, 8]. These cell types are distinguished by the expression of specific markers [7, 10, 11]. ISCs are irregularly arrayed across the basement membrane and surrounded by ECs and visceral muscle [8]. Previous studies have revealed age-related phenotypes of ISCs; ISC hyper-proliferation, DNA damage accumulation, and increased centrosome amplification are associated with intestinal hyperplasia [12–15]. ISC homeostasis (the balance of proliferation and quiescence) has been known to be regulated by extrinsic factors such as cytokine ligands of Janus kinase (JAK)–signal transducer and activator of transcription (STAT), epidermal growth factor receptor (EGFR), and wingless signaling that are derived from niches, including EC, EB, EE, and visceral muscle [16–20]. A recent study showed that hemocytes recruited to the midgut contribute to ISC homeostasis [21].

EC is an important niche in ISC homeostasis [18, 19, 22]. ECs are intestinal absorptive polyploid cells that constitute over 90% of the mass of *Drosophila* midgut [9]. Mature ECs are always exposed to external factors, owing to their location and functional properties. A well-known mechanism of EC in the field of ISC homeostasis is that the activation of c-Jun N-terminal kinase (JNK) in mature ECs induced by several external factors such as bacterial infection promotes ISC proliferation via paracrine signals derived from EC death [13, 18, 23]. The depletion of E-cadherin in ECs has been also reported to increase ISC proliferation [24]. However, more identification of the intrinsic factors in EC affecting ISC homeostasis is required for understanding of the mechanisms underlying the maintenance of ISC homeostasis

The EGFR/extracellular signal-regulated kinase signaling is a key pathway involved in intestinal homeostasis [19, 22, 23, 25, 26]. The Ras-Raf-MEK-ERK pathway is activated by transmitting signals recognized by EGFR [27, 28]. Previous studies revealed that EGFR/ERK signaling components are mainly detected in ISCs/EBs and that the signaling plays crucial roles for ISC proliferation and endoreplication during the differentiation of EBs to ECs [19, 25, 29]. In case of ECs, while EGFR/ERK components are silenced in mature ECs at the steady state, activation of EGFR/ERK signaling has been reported in response to external stress such as bacterial infection [23]. In addition, ERK signaling in ECs is known to be involved in antiviral defenses [30]. These facts indicate that EGFR/ERK signaling is tightly regulated in ECs for the maintenance of intestinal homeostasis. However, little is known about the regulation of EGFR/ERK signaling in EC as ISC niche.

Phosphatidylethanolamine-binding protein 1 (PEBP1)/Raf kinase inhibitory protein (RKIP) plays an important role as a physiological inhibitor of EGFR/ERK signaling pathway in mammals [31]. Loss or reduced expression of PEBP1/RKIP has been reported in aggressive cancer cells such as gastrointestinal tumor [32]. PEBP1 family is a highly conserved group of proteins in various species from bacteria to mammals [33–37]. *Drosophila* PEBP1 (CG18594), one of the six PEBPs, is highly expressed in larval and adult midgut [37–39], but its physiological function in the adult midgut remains unknown.

Here, we show that *Drosophila* PEBP1 plays a role as an EC-intrinsic suppressor of EGFR/ERK signaling and that the age-related decrease of PEBP1 in ECs accelerates ISC aging via EC death.

## RESULTS

### PEBP1 is strongly expressed in mature ECs of adult midgut

**Figure 1:**
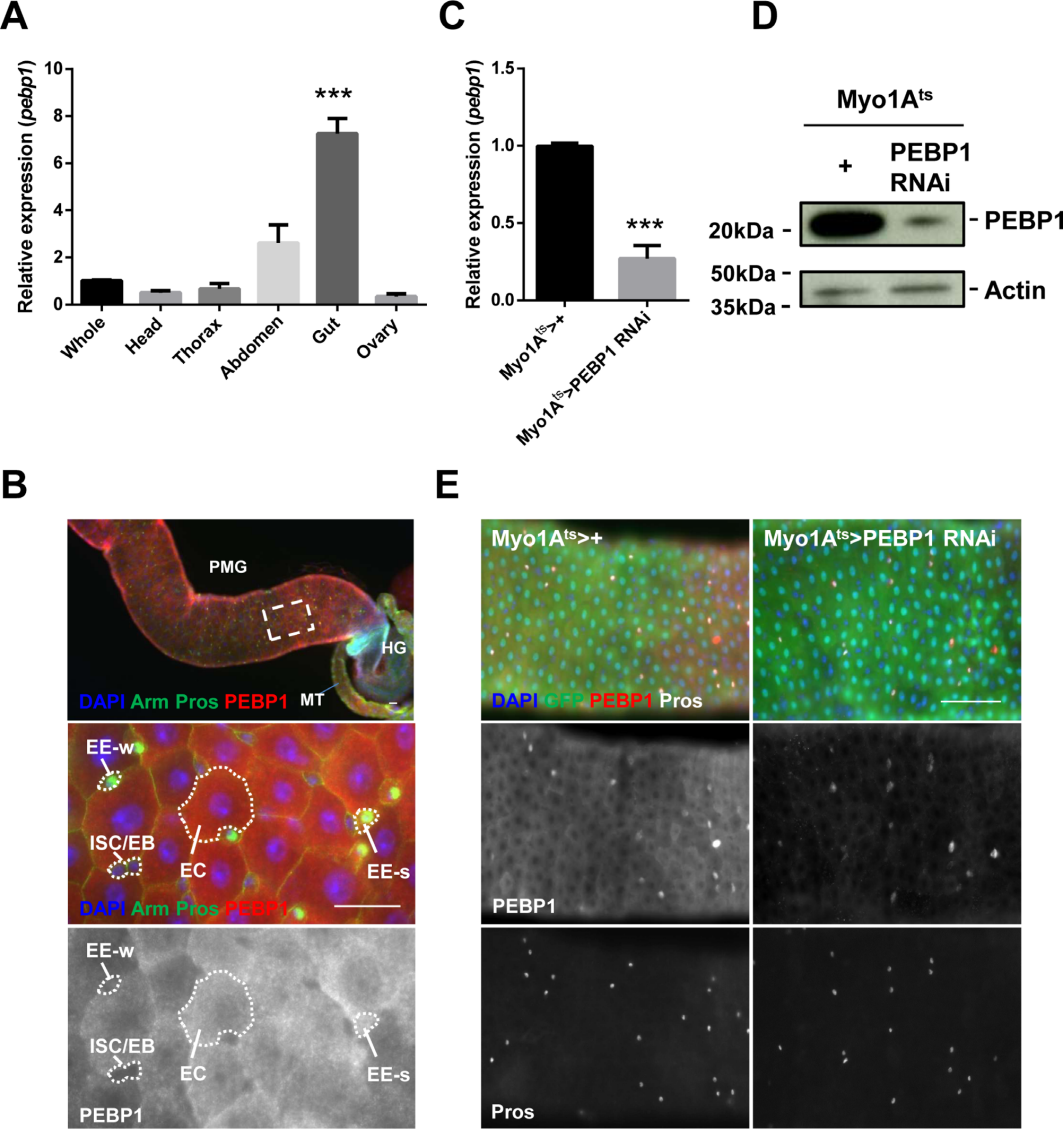
Expression of *Drosophila* PEBP1 in ECs of the adult gut (**A**) RT-qPCR analysis shows that the expression level of *pebp1* gene in the adult tissues. The expression level of *pebp1* mRNA was specifically higher in the gut as compared with other tissues. The expression levels of *pebp1* mRNA in various tissues were determined using extracts from the each tissues of 5-day-old OR flies. Means of five independent experiments ± SEM are shown. *P* values were calculated using a Student's *t*-test. ^***^p < 0.001 based on comparison to the whole body. (**B**) PEBP1 is expressed at a high level in the *Drosophila* midgut. The lower images are the enlarged images of the white dashed rectangle in upper panel. The expression of PEBP1 was weak in ISC/EB but strong in most ECs and some EEs (EE-s of lower panels). The guts from 5-day-old flies were stained with anti-Arm (green), anti-Pros (green), and anti-PEBP1 (red), and DAPI. PMG, posterior midgut. MT, Malpighian tubule. HG, hindgut. EE-s, EE-strong. EE-w, EE-weak. Scale bar, 20 μm. (**C**) RT-qPCR analysis of gut extracts shows that the expression level of *pebp1* gene decreased in EC-specific PEBP1 knockdown guts (*Myo1A^ts^ > PEBP1 RNAi*). Means of three independent experiments ± SEM are shown. *P* values were calculated using a Student's *t*-test. ^***^p < 0.001 based on comparison to the control guts. (**D**) Western blot analysis shows that the expression of PEBP1 significantly decreased in EC-specific PEBP1 knockdown guts as compared with those from control flies. Actin is used as loading control. (**E**) In EC-specific PEBP1 knockdown guts, the PEBP1 expression was specifically decreased in ECs. After 3 days of induction at 29° C, guts were stained with anti-GFP (green), anti-PEBP1 (red), anti-Pros (white), and DAPI (blue). Scale bar, 50 μm.

We investigated the expression of PEBP1 in *Drosophila* midgut by evaluating the transcriptional level of *pebp1* gene with RT-qPCR. PEBP1 expression was found to be higher in the gut as compared with other tissues (Figure 1A). Next, we determined the expression pattern of PEBP1 in gut cells using *Drosophila* anti-PEBP1 antibody constructed in the present study. The expression of PEBP1 was mainly detected in the midgut region (Figure 1B). A high level of PEBP1 expression was detected in the cytoplasm of almost all ECs and some EEs, while ISCs/EBs showed a weak expression of PEBP1 (Figure 1B). To further confirm the expression of PEBP1 in ECs, we constructed EC-specific PEBP1 knockdown strains using *Myo1A-Gal4;UAS-GFP,tub-Gal80^ts^* (*Myo1A^ts^)* which is active only in the mature EC [29]. After induction for 5 days at 29° C, the level of *pebp1* mRNA greatly decreased by 0.27-fold in EC-specific PEBP1 knockdown guts as compared with the control guts (Figure 1C). Moreover, a decrease in PEBP1 protein level was observed in EC-specific PEBP1 knockdown guts, as revealed by western blot analysis (Figure 1D). The EC-specific downregulation of PEBP1 was detected by immunostaining (Figure 1E). These results indicate that PEBP1 is highly expressed in the mature ECs of adult midgut.

### The decrease in PEBP1 expression is associated with increased ERK activity in ECs of aged guts

**Figure 2:**
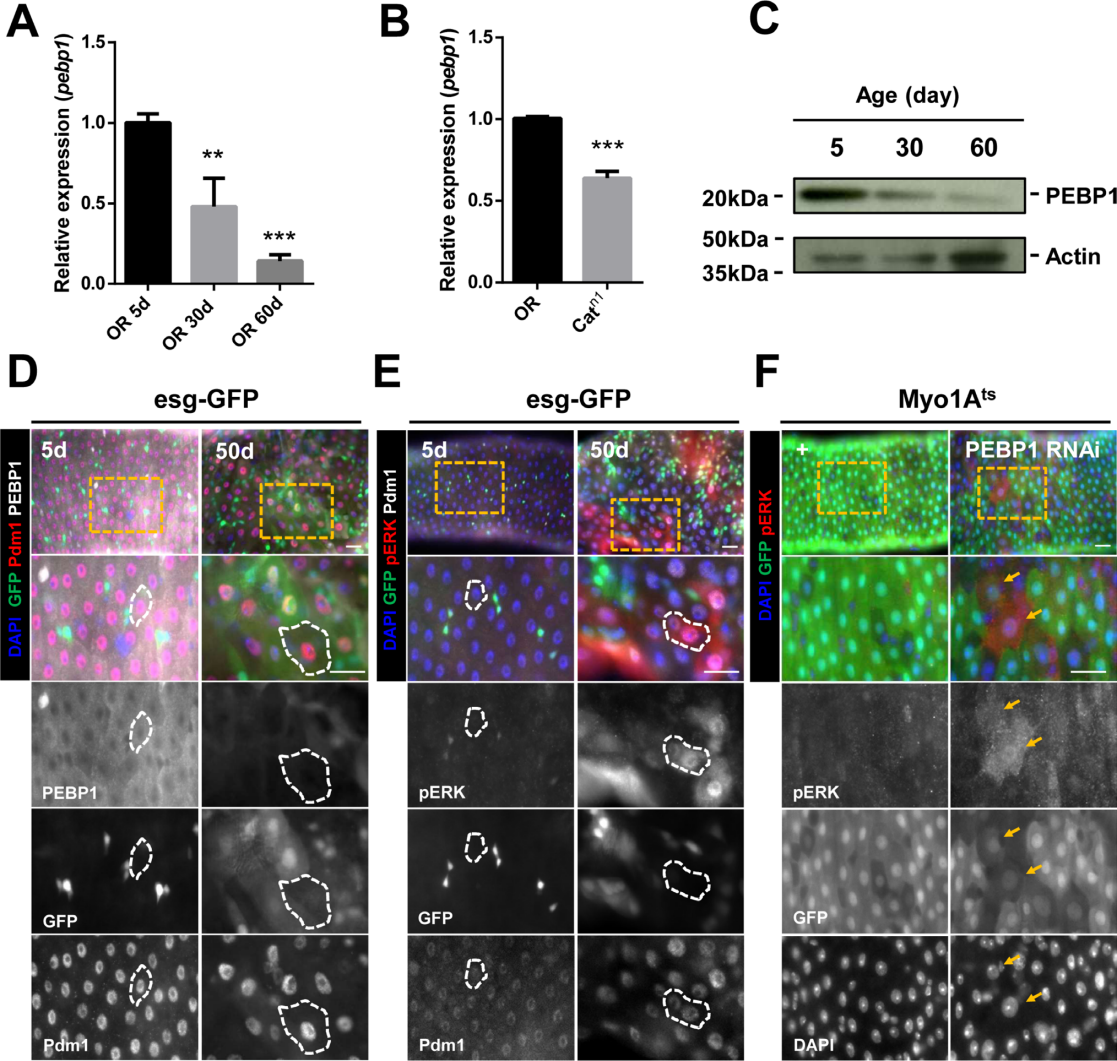
Decreased expression of PEBP1 is associated with increased ERK activity in ECs of aged midgut (**A**) RT-qPCR analysis shows that the expression level of *pebp1* mRNA decreased in the guts of 30- and 60-day old flies as compared with the guts from 5-day-old flies. The expression levels of PEBP1 was determined using extracts from guts of 5-, 30- or 60-day-old flies. Means of four independent experiments ± SEM are shown. *P* values were calculated using a Student's *t*-test. ^***^p < 0.001, ^**^p < 0.01 based on comparison to the 5-day-old flies. (**B**) RT-qPCR analysis shows that the expression level of *pebp1* mRNA was reduced in the guts of catalase mutant (*Cat^n1^/+*). The expression levels of PEBP1 were determined using extracts from the guts of 10-day-old OR and catalase mutant flies. Means of three independent experiments ± SEM are shown. *P* values were calculated using a Student's *t*-test. ^***^p < 0.001 based on comparison to the OR guts. (**C**) Western blot analysis shows that the protein level of PEBP1 decreased in the guts from 30- or 60-day-old flies as compared with those from 5-day-old flies. Actin is used as loading control. (**D**) PEBP1, which decreases in aged midguts, was observed in mature ECs (Pdm1^+^ and esg-GFP^−^ cells, white dashed line) using *esg-Gal4;UAS-GFP* (esg-GFP) fly strains. Guts of 5- and 50-day-old esg-GFP strains were stained with anti-GFP (green), anti-Pdm1 (red), anti-PEBP1 (white), and DAPI. The four lower panels are enlarged images of the yellow dashed rectangle in the upper panel. d, day. Scale bar, 20 μm (**E**) Increased expression of pERK was identified in mature ECs (Pdm1^+^ and esg-GFP^−^ cells, white line) of aged midgut. Guts of 5- and 50-day-old esg-GFP strains were stained with anti-GFP (green), anti-pERK (red), anti-Pdm1 (white), and DAPI (blue). The four lower panels are enlarged images of the yellow dashed rectangle in the upper panel. d, day. Scale bar, 20 μm. (**F**) ERK activity increased in ECs by EC-specific PEBP1 knockdown using *Myo1A^ts^*. After induction for 7 day at 29° C, guts of *Myo1A^ts^>+* or *Myo1A^ts^>PEBP1 RNAi* flies were stained with anti-pERK (red), anti-GFP (green), and DAPI. The four lower panels are enlarged images of the yellow dashed rectangle in the upper panel. Yellow arrows indicate ECs showing increased ERK activity (Myo-GFP^+^ and pERK^+^). d, day. Scale bar, 20 μm.

To evaluate whether the expression of PEBP1 is regulated by age, we determined the age-related changes in *pebp1* mRNA level in the gut. In comparison with the 5-day-old guts, 30- and 60-day-old guts showed a 0.48- and 0.14-fold decrease in the level of *pebp1* mRNA (Figure 2A). The decreased expression of *pebp1* mRNA was also detected in the guts of catalase mutant (*Cat^n1^/+*) flies used as the age-related oxidative stress mimic model [12] (Figure 2B). The age-related decrease in PEBP1 protein level in the midgut was confirmed using anti-PEBP1 antibody (Figure 2C). The number of ECs with reduced expression of PEBP1 (PEBP1^−^ and Pdm1^+^ cells) was increased in 30- and 60-day-old guts (Supplementary Figure 1A). Aged midguts showed large number of pre-mature polyploid cells formed by the mis-differentiation of EB, leading to a reduction in the proportion of mature ECs in total intestinal cells [12, 13]. To determine whether the age-related decrease in PEBP1 is associated with the reduced number of mature ECs in aged guts or decrease in mature ECs, we used *esg-Gal4;UAS-GFP* (*esg-GFP*) fly strains to distinguish mature polyploid ECs and mis-differentiated polyploid EC-like cells in aged guts. In comparison with the 5-day-old guts, the number of mature ECs with reduced expression of PEBP1 was increased in the 50-day-old guts (esg-GFP^−^ and Pdm1^+^, white dashed line) (Figure 2D). The reduction in PEBP1 expression was also detected in the ECs (Pdm1^+^) of catalase mutant flies (Supplementary Figure 1B). These results indicate the age-dependent increase in the frequency of mature ECs with reduced expression of PEBP1.

To evaluate whether *Drosophila* PEBP1 acts as a modulator of EGFR/ERK signaling, we investigated the correlation between PEBP1 expression and ERK activity in ECs using anti-phospho p44/p42 (pERK1/2) antibody as a marker for EGFR/ERK signaling [40]. We found that the number of ECs expressing pERK (pERK^+^ and Pdm1^+^ cells) was increased in 30-day-old and 60-day-old midguts (Supplementary Figure 1C, arrows). In addition, the number of mature ECs expressing pERK (esg-GFP^−^ and Pdm1^+^ cells, white line of right panels) was increased in 50-day-old guts of *esg-GFP* fly strain, in comparison with 5-day-old guts (Figure 2E). The increase number of ECs expressing pERK was also found in the guts of catalase mutant flies and paraquat (PQ), as an oxidative stress inducer [12, 41], treated flies (Supplementary Figure 1D and Supplementary Figure 2A). Furthermore, EC-specific PEBP1 knockdown guts showed an increase pERK^+^ ECs as compared with control guts (Figure 2F; Myo-GFP^+^ cells, yellow arrows). These results show that the downregulation of PEBP1 is related with an increase in ERK activity in ECs of aged guts.

To confirm that the PEBP1 is a modulator of EGFR/ERK signaling in ECs, we investigated whether overexpression of PEBP1 in ECs can regulate ERK signal under PQ treatment using *Myo1A^ts^ >+* and *Myo1A^ts^ > PEBP1* flies. After 18h PQ treatment, the number of pERK^+^ ECs was increased in the guts of control flies (*Myo1A^ts^ >+*), while the overexpression of PEBP1 in ECs reduced the number of pERK^+^ ECs (Supplementary Figure 2B and 2C). This results suggest that PEBP1 act as an EC-intrinsic suppressor of EGFR/ERK signaling.

### Downregulation of PEBP1 in ECs causes age-related phenotypes in ISCs

**Figure 3:**
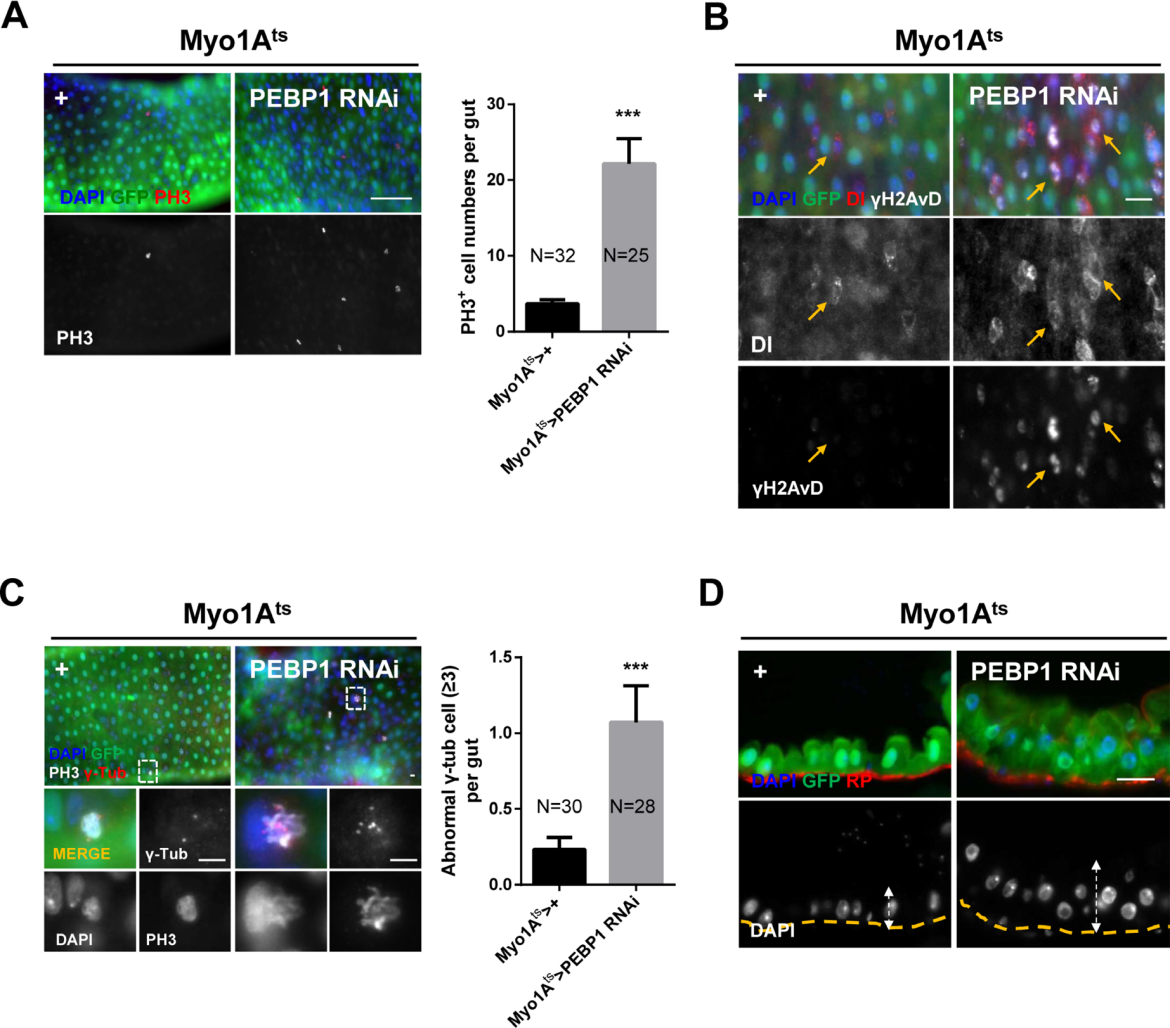
EC-specific knockdown of PEBP1 causes age-related phenotypes of ISCs (**A**) Mitotic index of ISCs increased by EC-specific PEBP1 knockdown using *Myo1A^ts^*. After induction for 7 days at 29° C, guts of *Myo1A^ts^>+* or *Myo1A^ts^>PEBP1 RNAi* flies were stained with anti-GFP (green), anti-PH3 (red) and DAPI (blue). The number of PH3^+^ cells was counted in whole guts. Data (mean ± SEM) collated from 25–32 guts. N: number of counted guts. *P* values were calculated using a Student's *t*-test. ^***^p < 0.001 based on comparison to the control guts. Scale bar, 50 μm. (**B**) Gamma-H2AvD accumulation was observed in ISCs by EC-specific PEBP1 knockdown. After induction for 7 days at 29° C, guts of *Myo1A^ts^>+* or *Myo1A^ts^>PEBP1 RNAi* flies were stained with anti-GFP (green), anti-γH2AvD (white), anti-Dl (red) and DAPI (blue). Yellow arrows indicate ISCs (Dl^+^ cells). Scale bar, 20 μm. (**C**) The numbers of mitotic ISCs with supernumerary centrosome (≥3) were increased by EC-specific PEBP1 knockdown. After induction for 7 days at 29° C, guts of *Myo1A^ts^>+* or *Myo1A^ts^>PEBP1 RNAi* flies were stained with anti-GFP (green), anti-PH3 (red), anti-γ-Tubulin (γ-Tub, white), and DAPI (blue). Lower four panels are magnified images of the white dashed rectangle in the upper panels. The numbers of mitotic ISCs with supernumerary centrosomes (≥3) were counted in whole guts. Data (mean ± SEM) collated from 28–30 guts. N, number of counted guts. *P* values were calculated using a Student's *t*-test. ^***^p < 0.001 based on comparison to the control guts. Scale bar, 5 μm. (**D**) Sagittal view of midgut epithelium hyperplasia induced by EC-specific PEBP1 knockdown. After induction for 7 days at 29° C, guts of *Myo1A^ts^>+* or *Myo1A^ts^>PEBP1 RNAi* flies were stained with anti-GFP (green), rhodamine phalloidin (RP, red) and DAPI (blue). Yellow lines indicate RP of upper panels and white arrows indicate thickness. Original magnification is 400×. Scale bar, 20 μm.

To assess whether the downregulation of PEBP1 in ECs affects ISC homeostasis, we characterized the phenotypes of ISCs by EC-specific knockdown of PEBP1 under *Myo1A^ts^*. We first evaluated the proliferation of ISCs using anti-phospho-histone H3 (PH3) antibody as a mitosis marker. After 7 days of induction at 29° C, the number of PH3^+^ cells increased by up to 6.03-fold in EC-specific PEBP1 knockdown guts as compared with the control guts (Figure 3A), indicative of ISC hyper-proliferation induced by PEBP1 depletion. As hyper-proliferation is linked to DNA damage accumulation and centrosome amplification in aged ISCs [14, 15], we assessed whether the depletion of PEBP1 in ECs induced DNA damage accumulation and centrosome amplification in ISCs. We examined the expression of γH2AvD, the *Drosophila* ortholog of γH2AX and a marker of DNA double-strand break, to evaluate DNA damage accumulation in ISCs. The level of γH2AvD increased in ISCs marked with Dl^+^ (Figure 3B). Furthermore, the number of mitotic ISCs with supernumerary centrosomes was significantly increased in the midguts of EC-specific PEBP1 knockdown flies (Figure 3C). ISC hyper-proliferation has been known to be associated with intestinal hyperplasia [13]. EC-specific knockdown of PEBP1 induced hyperplasia with abnormal multi-layer epithelium (Figure 3D). These results clearly indicate that the decrease in PEBP1 level in ECs induces age-related phenotypes in ISCs.

### Downregulation of PEBP1 in ECs accelerates ISC aging via EC death

**Figure 4:**
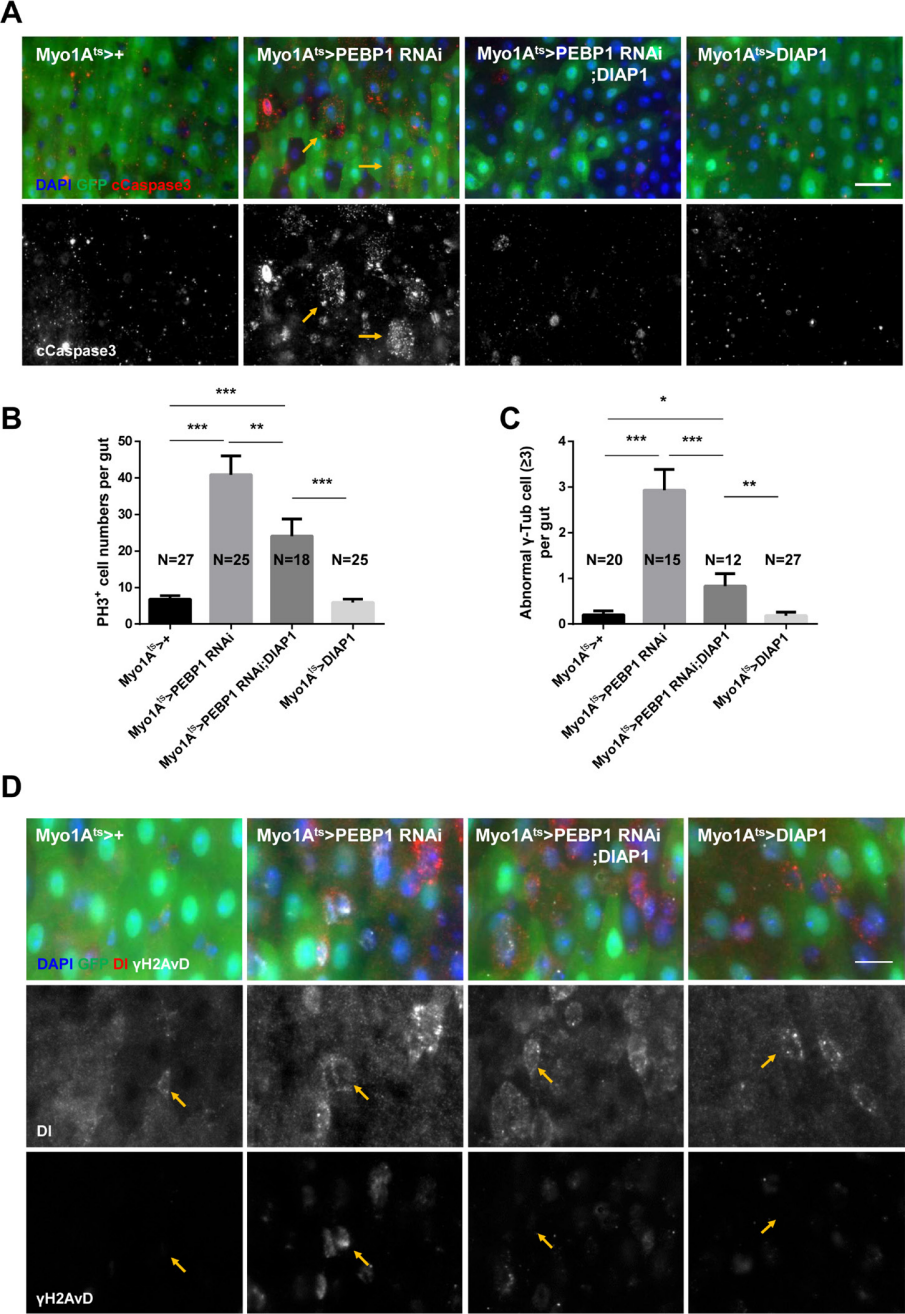
EC-specific knockdown of PEBP1 causes age-related phenotypes of ISCs via EC death (**A**) An increase in the number of cleaved caspase-3 in EC-specific PEBP1 knockdown guts was compensated by EC-specific DIAP1 overexpression. After induction for 7 days at 29° C, guts of *Myo1A^ts^>+*, *Myo1A^ts^>PEBP1 RNAi, Myo1A^ts^>PEBP1 RNAi;DIAP1 or Myo1A^ts^>DIAP1* flies were stained with anti-GFP (green), anti-cleaved caspase-3 (cCapase3, red) and DAPI (blue). Scale bar, 20 μm. (**B**) The increased ISC mitotic index of EC-specific PEBP1 knockdown guts was compensated by EC-specific DIAP1 overexpression. After induction for 7 days at 29° C, guts were stained with anti-GFP (green), anti-PH3 (red) and DAPI (blue). The number of PH3^+^ cells was counted in whole guts of EC-specific PEBP1 knockdown flies. Data (mean ± SEM) were collated from 18–27 guts. N, number of counted guts. *P* values were calculated using a Student's *t*-test. ^***^p < 0.001, ^**^p < 0.01 (**C**) The increase in the number of mitotic ISCs with supernumerary centrosome (≥3) in EC-specific PEBP1 knockdown guts was compensated by EC-specific DIAP1 overexpression. After induction for 7 days at 29° C, guts were stained with anti-GFP (green), anti-γ-Tubulin (γ-Tub, white), anti-PH3 (red) and DAPI (blue). Number of γ-Tubulin signal was counted in mitotic ISC of whole guts. Data (mean ± SEM) were collated from 12–27 guts. N, number of counted guts. *P* values were calculated using a Student's *t*-test. ^***^p < 0.001, ^**^p < 0.01, ^*^p < 0.05. (**D**) Increased γH2AvD accumulation of ISCs in EC-specific PEBP1 knockdown guts was compensated by EC-specific DIAP overexpression. After induction for 7 days at 29° C, guts were stained with anti-GFP (green), anti-Dl (red), anti-γH2AvD (white), and DAPI (blue). Yellow arrows indicate ISCs (Dl^+^ cells). Original magnification is 400×. Scale bar, 10 μm.

It is known that EC death is a common niche phenomenon promoting ISC proliferation through the secretion of several cytokines [18]. As the decrease in PEBP1 induced ISC hyper-proliferation, we assessed whether the downregulation of PEBP1 induces EC death using cleaved caspase-3 antibody. We observed an increase in the number of cleaved caspase-3^+^ ECs in EC-specific PEBP1 knockdown guts as compared to controls (Figure 4A). This result indicates that the downregulation of PEBP1 in ECs induces EC death, suggestive of the protective role of PEBP1 against EC death. We explored the protective role of PEBP1 against EC death using ionizing radiation, a valuable tool to induce cell death [42], and conducted EC-specific overexpression of PEBP1 in the fly strain. After 20 Gy irradiation, the number of cleaved caspase-3^+^ ECs was significantly increased in the guts of control flies, while the overexpression of PEBP1 in ECs reduced the number of cleaved caspase-3^+^ ECs by irradiation (Supplementary Figure 3). This result indicates that the expression level of PEBP1 is associated with EC death.

We evaluated whether the age-related phenotypes in ISCs following EC-specific knockdown of PEBP1 was associated with EC death. We constructed PEBP1 knockdown strain with concomitant overexpression of *Drosophila* inhibitor of apoptosis 1 (DIAP1). The increase in the number of cleaved caspase-3^+^ ECs by PEBP1 knockdown was rescued by DIAP1 overexpression (Figure 4A). Furthermore, the increased proliferation and centrosome amplification in ISCs in response to EC-specific PEBP1 knockdown were compensated by DIAP1 overexpression (Figure 4B and 4C). Moreover, the increased γH2AvD accumulation was also compensated under same conditions (Figure 4D). These results indicate that the decreased level of PEBP1 in ECs induces age-related phenotypes in ISCs and intestinal hyperplasia via EC death.

### Inhibition of ERK activity rescues the phenotypes induced by PEBP1 depletion

As the decreased expression of PEBP1 was closely related with the increase in ERK activity in ECs of aged guts (Figure 2), we assessed whether the phenotypes mediated by EC-specific PEBP1 knockdown were associated with the increased ERK activity in ECs. We investigated the phenotypes by EC-specific overexpression of activated Raf using *Myo1A^ts^* and *UAS-Raf^gof^* (Raf gain of function). The EC-specific overexpression of activated Raf in the guts resulted in an increase in the activity of ERK accompanied with the increased number of cleaved caspase-3^+^ ECs, enhanced proliferation, centrosome amplification, DNA damage accumulation in ISCs, and intestinal hyperplasia (Supplementary Figure 4). These results indicate that the increased Raf/ERK signaling activity in ECs causes EC death, age-related phenotypes in ISCs, and intestinal hyperplasia.

**Figure 5:**
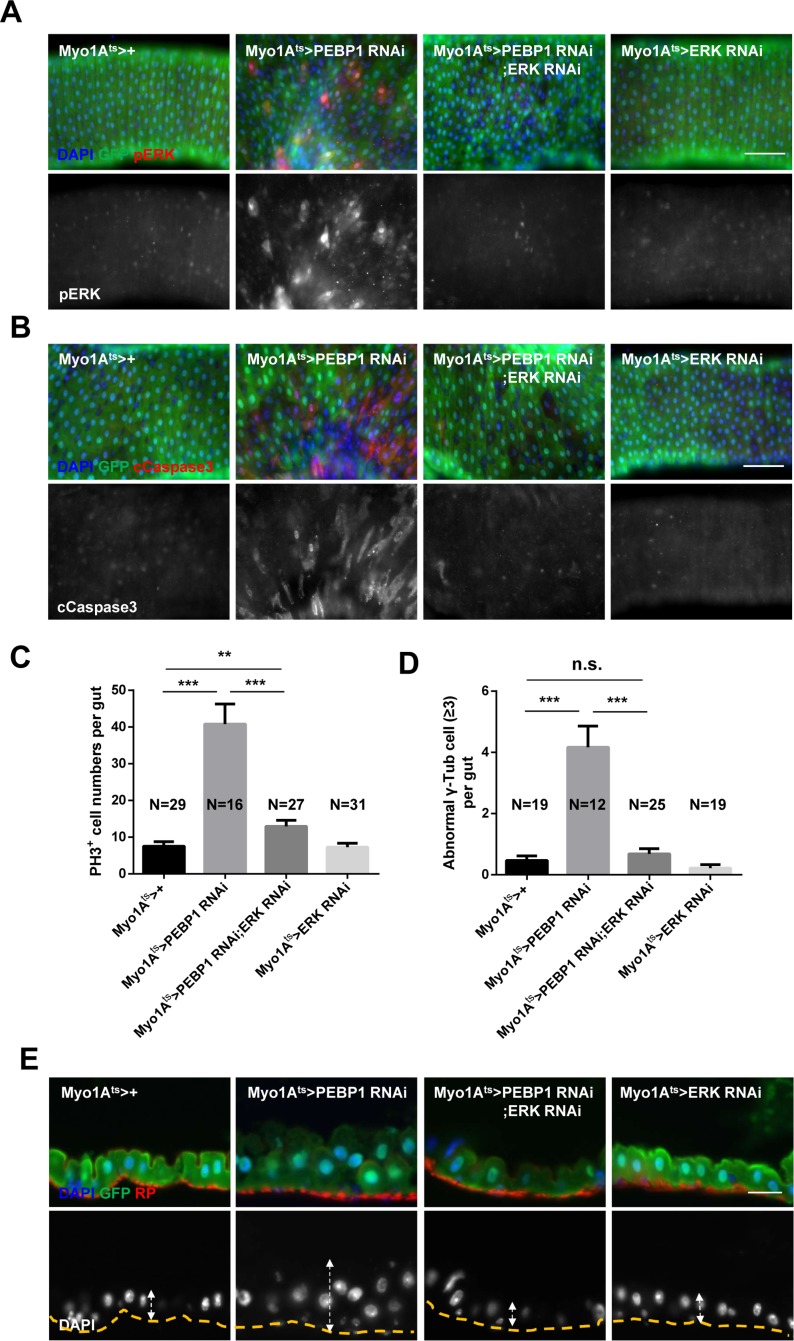
Inhibition of ERK activity rescues the phenotypes induced by PEBP1 depletion (**A**) The increased level of ERK in ECs of EC-specific PEBP1 knockdown guts was reduced by EC-specific ERK knockdown. After induction for 7 days at 29° C, guts of *Myo1A^ts^>+*, *Myo1A^ts^>PEBP1 RNAi, Myo1A^ts^>PEBP1 RNAi;ERK RNAi* or *Myo1A^ts^>ERK RNAi* were stained with anti-GFP (green), anti-pERK (red) and DAPI (blue). Original magnification is 400×. Scale bar, 20 μm. (**B**) The increase in the number of cleaved caspase-3^+^ ECs in EC-specific PEBP1 knockdown guts was compensated by EC-specific ERK knockdown. After induction for 7 days at 29° C, guts were stained with anti-GFP (green), anti-cleaved caspase-3 (cCapase3, red) and DAPI (blue). Scale bar, 50 μm. (**C**) Increased mitotic index of EC-specific PEBP1 knockdown guts was reduced by EC-specific ERK knockdown. After induction for 7 days at 29° C, guts were stained with anti-GFP (green), anti-PH3 (red) and DAPI (blue). The number of PH3^+^ cells was counted in whole guts. Data (mean ± SEM) collated from 16–31 guts. N, number of counted guts. *P* values were calculated using a Student's *t*-test. ^***^p < 0.001, ^**^p < 0.01. (**D**) The increase in the mitotic ISCs with supernumerary centrosome (≥3) of EC-specific PEBP1 knockdown guts was reduced by EC-specific ERK knockdown. The number of γ-Tubulin signal was counted in mitotic ISC of whole guts. Data (mean ± SEM) were collated from 12–25 guts. N, number of counted guts. *P* values were calculated using a Student's *t*-test. ^***^p < 0.001, ^**^p < 0.01, ^*^p < 0.05, n.s. not significant. (**E**) Midgut epithelium hyperplasia induced by EC-specific PEBP1 knockdown was compensated by EC specific ERK knockdown. After induction for 7 days at 29° C, guts were stained with anti-GFP (green), RP (red) and DAPI (blue). The yellow lines indicate RP of upper panels and white arrows indicate thickness. Original magnification is 400×. Scale bar, 20 μm.

To explore the direct association between EC-specific PEBP1 depletion and increased ERK activity in EC death and ISC aging, we examined whether the phenotypes induced by EC-specific PEBP1 knockdown could be rescued by the inhibition of ERK activity. RNAi-mediated downregulation of ERK has been reported to reduce ERK signaling activity [30], as confirmed in the present study (Supplementary Figure 5). Following induction for 7 days at 29° C, the increase in the number of pERK^+^ EC and cleaved caspase-3^+^ ECs induced by EC-specific knockdown of PEBP1 was compensated with EC-specific knockdown of ERK (Figure 5A, 5B and Supplementary Figure 6). The increased mitotic index and DNA damage accumulation in ISCs induced by EC-specific knockdown of PEBP1 were also recovered by EC-specific knockdown of ERK (Figure 5C and Supplementary Figure 7A). Furthermore, the increase in centrosome amplification and hyperplasia phenotypes was compensated following EC-specific knockdown of ERK (Figure 5D and 5E and Supplementary Figure 7B). Thus, EC death and age-related phenotypes of ISCs induced by EC-specific knockdown of PEBP1 are related to the increase in ERK signaling in ECs and PEBP1 acts as an inhibitor of ERK signaling in ECs.

## DISCUSSION

Here, we established, in the adult *Drosophila* midgut system, the age-related downregulation of PEBP1 in ECs is a novel intestinal niche factor accelerating ISC aging. We also found that PEBP1 acts as an inhibitor of EGFR/ERK signaling

The present study evidences that PEBP1 acts as an EC-intrinsic suppressor of ERK signaling. While the involvement of EGFR/ERK signaling in ISC homeostasis has been well defined [19, 20, 25], a better understanding of the regulation of EGFR/ERK, especially in niches, is required with regard to the involvement of dysregulated EGFR/ERK signaling in tumorigenesis. Under normal physiological condition, EGFR/ERK signaling components are mainly expressed in ISCs/EBs and weakly in mature ECs [19, 29]. Here, we showed that PEBP1 is weekly expressed in ISCs/EBs and highly in mature ECs. We observed an increase in ERK activity by the downregulation of PEBP1 and that the inhibition of ERK activity rescued the phenotypes induced by PEBP1 depletion. These data indicate that PEBP1 acts as an inhibitor of ERK signaling in mature ECs. In mammals, PEBP1/RKIP is known as an important regulator for suppressing MAPK signaling pathway by binding to Raf, MAPK, or ERK [31, 43, 44]. *Drosophila* PEBP1 harbors the complete consensus signature for PEBP family (D-P-D-xP-x(11)-H-x(28, 30)-H-R), which is a part of the ligand-binding pocket [45], although PEBP1 only share approximately 40% identity with mammalian PEBPs [39]. Thus, our data suggest that *Drosophila* PEBP1 may be functionally similar to the mammalian PEBP1/RKIP, although it is still unclear how *Drosophila* PEBP1 negatively contribute to ERK signaling.

We also established that the downregulation of PEBP1 in ECs is a novel cause accelerating ISC aging. In the present study, the downregulation of PEBP1 and Raf overexpression in ECs induced age-related phenotypes of ISCs such as hyperproliferation, DNA damage accumulation and the increase in centrosome amplification, and hyperplasia. Our study also revealed that EC-specific depletion of PEBP1 induces EC death and our data in the experiment with concomitant DIAP1 overexpression revealed that the age-related phenotypes of ISCs by the downregulation of PEBP1 are associated with EC death. It is known that EC death is a common niche phenomenon promoting ISC proliferation through the secretion of several cytokines [18]. Increased ERK activity in ECs has been reported to cause death of ECs [19, 23]. It was also reported that the increase in ERK activity in ECs induces the expression of Upd3 in ECs, leading to ISC hyper-proliferation [19]. Whether the phenotypes of ISCs induced by decreased PEBP1 in ECs are related with these cytokines is still unclear; however, our data indicate that the age-related decrease in PEBP1 in ECs accelerates ISC aging via EC death, suggesting that PEBP1 plays an anti-apoptotic role in EC. Our data imply the involvement of misregulated EGFR/ERK signaling in niche in ISC aging and the importance of negative regulator of EGFR/ERK signaling in niche for ISC homeostasis. To date, little is known about the role of PEBP1-ERK signaling in stem cell behavior. Our study shows for the first time the role of PEBP1 in the field of adult stem cell proliferation and aging. Because the expression of PEBP1 is detected in most tissues [37, 46, 47], it would be interesting to survey the role of PEBP1-ERK signaling in other stem cells.

Here, we evidence that the age-related downregulation of negative regulator is a cause of misregulated EGFR/ERK signaling during aging. In this study, we showed the activation of ERK in ECs of aged guts. We also showed the downregulation of PEBP1 in aged guts, indicating the age-related downregulation of negative regulator of EGFR/ERK signaling. In mammals, aberrant ERK signaling during aging has been known [48, 49]. The outcome of abnormal activation of ERK signaling is shown to accelerate tumorigenesis [50–52]. The decreased expression of PEBP1/RKIP has been reported in many types of cancer cells and malignant tissues [32, 53–55]. The reduction in PEBP1/RKIP was also reported in aged neuron and skin [56, 57], although increased PEBP1/RKIP expression in inflammatory bowel disease was recently reported [58]. Our data, together with those found similar evidences, indicate that the age-related downregulation of PEBP1 may well be conserved from the fly to humans. At present, the mechanisms underlying age-related downregulation of PEBP1 are unclear. Oxidative stress/reactive oxygen species (ROS) is considered a major cause of mechanisms underlying tissue aging and the pathogenesis of various age-related diseases [59]. ROS have been known to induce EGFR/ERK activation during aging [60, 61]. Here, we showed the activation of ERK and the downregulation of PEBP1 in ECs of oxidative stressed guts using catalase mutant flies. Taken together, our data suggest that downregulation of PEBP1 likely causes dysregulated EGFR/ERK signaling during aging, revealing how ROS mediates the activation of ERK in the aging. Our data also suggest that redox-sensitive regulator system including transcription factor might be involved in the regulation of PEBP1 gene.

In summary, our data strongly indicate that strong expression of PEBP1 in mature ECs of adult midgut is required for ISC homeostasis as EC-intrinsic inhibitor of EGFR/ERK signaling. Our study presents evidence showing that the age-related downregulation of PEBP1 in ECs is a novel cause accelerating ISC aging. In addition, our data provide molecular insight into the tight regulation of ERK signaling for intestinal homeostasis during aging.

## MATERIALS AND METHODS

### Fly stocks and culture conditions

All *Drosophila* stocks were maintained on standard cornmeal-sugar-yeast medium at 25° C under a 12 h light/dark cycle, as previously described [12]. The medium comprised 79.2% water, 1% agar, 7% cornmeal, 2% yeast, 10% sucrose, 0.3% bokinin, and 0.5% propionic acid. To avoid larval overpopulation in all vials, 50–60 adult flies per vial were transferred to new food vials every 2−3 days throughout the experiment. In all experiments in this study, adult female flies were used, and *Oregon-R* (OR) flies were used as wild-type. Catalase mutant (*Cat^n1^/+*) flies were obtained from a cross of OR males and *Cat^n1^/TM3* females. *Delta-Gal4* and *tubulin-Gal80* fly strains were combined to the different chromosome to generate teperature inducible *tubulin-Gal80^ts^;Delta-Gal4* (*Dl^ts^*) flies. For the induction of gene expression, conditional expression in adult flies using *tub-Gal80^ts^* was achieved by maintaining flies at 18° C throughout their developmental stage and shifting the 3-day-old flies to a higher temperature (29° C) for 3, 5, or 7 days. *Esg-Gal4;UAS-GFP (esg-GFP*) were obtained from B. Ohlstein [10]. *Myo1A-Gal4;UAS-GFP,tub-Gal80^ts^* (*Myo1A^ts^*) were obtained from B. A. Edgar [18]. *Delta-Gal4* were obtained from S. X. Hou [62]. *UAS-PEBP1 RNAi* (#20650, #101957) and *UAS-ERK RNAi* (#35641) strains were obtained from the Vienna Drosophila RNAi Center (VDRC, Vienna, Austria). *UAS-PEBP1* (#35835), *UAS-Raf^gof^* (#2033), *UAS-DIAP1* (#6657), and *tub-Gal80^ts^* (#7108) strains were obtained from the Bloomington Drosophila Stock Center (BDSC, Indiana University, Bloomington, IN, USA).

### Reverse transcription quantitative polymerase chain reaction (RT-qPCR)

Total RNA was isolated from adult tissues using TRIzol reagent (Molecular Research Center, Cincinnati, OH, USA) according to the manufacturer's instructions, and 1 μg total RNA was reverse transcribed using M-MLV reverse transcriptase (Promega, Madison, WI, USA). Quantitative real-time PCR was performed using iQ^TM^ SYBR^®^ Green Supermix (Bio-Rad, Hercules, CA, USA). Data were acquired on a DNA Engine Chromo4 instrument (Bio-Rad). Cycling conditions were 30 s at 95° C, 30 s at 52° C, and 30 s at 72° C. Products were analyzed via melting curve analysis for 10 s at 95° C and 15 s at 65° C, followed by a temperature ramp from 65 to 95° C (0.1° C/s) and continuous fluorescence recording. Oligonucleotide sequences for the analysis of *pebp1* [45] and *rp49* [13] were previously reported.

### Immunohistochemistry

Immunostaining was performed as previously described [14, 19]. Intact adult guts were dissected and fixed for 30 min in 1×phosphate-buffered saline (1×PBS) containing 4% paraformaldehyde (Electron Microscopy Sciences, Hatfield, PA, USA), dehydrated for 5 min in 50%, 75%, 87.5%, and 100% methanol, and rehydrated for 5 min in 50%, 25%, and 12.5% methanol in PBST (0.1% Triton X-100 in 1×PBS). After washing in 1×PBS/1×PBST, samples were incubated overnight with primary antibodies in 1% bovine serum albumin (BSA; Sigma-Aldrich, St. Louis, MO, USA) at 4° C. The samples were washed in PBST and incubated with secondary antibodies at 25° C for 60 min. Following incubation, the samples were washed and mounted with Vectashield (Vector Labs, Burlingame, CA, USA). Images were analyzed using a Zeiss AxioSkop 2 Plus microscope (Carl Zeiss Inc., Jena, Germany).

### Cryosections

Midguts were dissected, fixed in 4% paraformaldehyde at room temperature for 30 min, and infiltrated overnight with 20% sucrose at 4° C. After flash-freezing in Tissue-Tek optimal cutting temperature (OCT) medium (SAKURA, Tokyo, Japan), 10 μm sections were cut on a cryostat (Leica CM1850; Leica Microsystems, Wetzlar, Germany) at −20° C. Sections were blocked in 5% BSA (Sigma-Aldrich) for 1 h and incubated overnight with primary antibodies. The samples were washed in PBST and incubated with secondary antibodies conjugated to a fluorescent dye at 25° C for 1 h, followed by their mounting with Vectashield (Vector Labs) and analysis using a Zeiss AxioSkop 2 Plus microscope (Carl Zeiss Inc.).

### Antibodies

For the immunohistochemistry, the following primary antibodies were used at indicated dilutions: mouse anti-Delta (Dl, ISC marker), 1:100, mouse anti-Prospero (Pros, EE marker), 1:100 and mouse anti-Armadillo (Arm), 1:100 (Developmental Studies Hybridoma Bank, IA, USA); mouse anti-GFP and rabbit anti-GFP, 1:500 (Molecular Probes, Eugene, OR, USA); rat anti-GFP, 1:500 (Nacalai Tesque, Kyoto, Japan); rabbit anti-γH2AvD, 1:1000 (Rockland, Gilbertsville, PA, USA); mouse anti-γ-tubulin, 1:500 (Sigma-Aldrich); rabbit anti-phospho-histone H3, 1:500 (PH3, Millipore, Billerica, MA, USA); rabbit anti-cleaved caspase-3, 1:100 and rabbit anti-p44/p42 mitogen-activated protein kinase (MAPK; anti-pERK), 1:100 (Cell Signaling Technologies, Danvers, MA, USA); mouse anti-Pdm1, 1:100 (EC marker, from S.M. Cohen); rabbit anti-Pdm1, 1:100 (from Y. Cai); and rabbit anti-PEBP1, 1:200 (developed in this study). The following secondary antibodies were used at indicated dilutions: fluorescein isothiocyanate (FITC)-conjugated goat anti-rabbit, 1:500, Cy3-conjugated goat anti-rabbit, 1:500, FITC-conjugated goat anti-mouse, 1:500 and Cy3-conjugated goat anti-mouse, 1:500 (Jackson ImmunoResearch, West Grove, PA, USA). Nuclei were labeled with 4,6-diamidino-2-phenylindole (DAPI), 1:500 (Molecular Probes). Visceral muscle (VM) was labeled with rhodamine phalloidin (RP), 1:500 (Life Technologies, Grand Island, NY, USA).

### PEBP1 antibody production

*Drosophila* PEBP1 (CG18594) antibody was produced by NovoPro Bioscience Inc. (Shanghai, China). PEBP1 peptide was synthetized by solid phase using Fmoc chemistry. The purity and amino acid composition were confirmed by high-performance liquid chromatography (HPLC) and electrospray ionization mass spectrometry (ESI-MS). The peptide (DQPTVVFDAEPNSLY-Cys) was coupled to keyhole limpet hemocyanin (KLH) through thiol. KLH-coupled antigens (about 1 mg) were mixed with an equal volume of complete Freund adjuvant and injected subcutaneously into two New Zealand rabbits. Boosts (0.5 mg) were given 2 weeks later at an interval of 1 week. Immunopositive sera were monitored by enzyme-linked immunosorbent assay (ELISA) with KLH-coupled antigens as screening peptides. After seven boosts (about 9 weeks), the anti-serum was purified with a peptide-affinity column.

### Western blot analysis

A total of 30 guts were dissected from 5-, 30- and 60-day-old flies, or *Myo1A^ts^>+* and *Myo1A^ts^>PEBP1 RNAi* flies after induction of 5 days at 29° C. Total protein from these guts were extracted using a lysis buffer (Intron Biotechnology, Korea) with a protease inhibitor (Roche, Basel, Switzerland). Protein quantification was done with Bradford reagent (Bio-Rad) before equal amount of protein was loaded. The whole gut extracts were separated by sodium dodecyl sulfate (SDS)-polyacrylamide gels containing 15% acrylamide and the protein bands were transferred onto polyvinylidene fluoride membranes (GE Healthcare Life Sciences, England). The blotted membranes were blocked with 1×TBST containing 5% skim milk, followed by their overnight incubation with the rabbit PEBP1 antibody (1:2000) and goat anti-actin antibody (1:5000; Santa Cruz Biotechnology, Dallas, TX, USA) at 4° C. After washing, the membranes were incubated with horseradish peroxidase (HRP)-conjugated secondary antibodies (1:5000) for 2 h at room temperature. The protein bands were detected with enhanced chemiluminescence (ECL) western blotting detection reagent (ThermoFisher Scientific, MA, USA).

### Paraquat assay

Paraquat (N,N′-dimethyl-4,4′-bipyridinium dichloride, PQ) feeding assay was conducted as previously described [12]. Five-day-old OR flies, or *Myo1A^ts^>+* and *Myo1A^ts^>PEBP1* flies after induction of 5 days at 29° C were starved for 2 h and exposed to 10 mM paraquat (Sigma-Aldrich) in 1% sucrose media for 18 h. During paraquat treatment, *Myo1A^ts^>+* and *Myo1A^ts^>PEBP1* flies were maintained in a 29° C incubator for the activation of the gene expression of UAS-Gal4 system. Following paraquat treatment, guts were subjected to immunostaining.

### Ionizing radiation

Flies were irradiated with a single dose of 20 Gy using a gamma irradiation machine (137Cs, 21.275tBq [575Ci], MDS Nordion International, Ottawa, Canada) at a dose rate of 2.25 Gy/min. A continuous dose was administered to the whole body of flies in the irradiation chamber at room temperature. After irradiation, irradiated flies were maintained in a 29° C incubator for the activation of the gene expression of UAS-Gal4 system.

### Quantitative analyses

The numbers of PH3^+^ cells and supernumerary centrosomes (≥3) in mitotic ISCs were determined in whole midguts, defined from R1 to R5 by Buchon *et al* [63]. The numbers of cleaved caspase-3^+^ ECs were counted from the posterior midgut R5 region under microscopic fields at a magnification of 400×. Data are expressed as mean ± standard error of the mean (SEM) and the statistical significance of differences was determined using the Student's *t*-test (unpaired, two-sided *t*-test). A value of *p* < 0.05 was considered statistically significant. GraphPad Prism 6 (GraphPad Software, La Jolla, CA, USA) was used for the analysis of variance.

## SUPPLEMENTARY MATERIALS FIGURES AND TABLES



## Abbreviations

DIAP1: *Drosophila* inhibitor of apoptosis 1
EB: enteroblast
EC: enterocyte
EE: enteroendocrine cell
EGFR: epidermal growth factor receptor
ERK: extracellular signal-regulated kinase
ISC: intestinal stem cell
JAK: Janus kinase
JNK: c-Jun N-terminal kinases
PEBP1: phosphatidylethanolamine binding protein 1
PQ: paraquat
RKIP: Raf kinase inhibitory protein
ROS: reactive oxygen species
STAT: signal transducer and activator of transcription.
