# Supplementary material for: Drosophila PEBP1 inhibits intestinal stem cell aging via suppression of ERK pathway
Source: Oncotarget. 2018 Apr 6;9(26):17980–93. doi: 10.18632/oncotarget.24834 (PMC5915051; doi:10.18632/oncotarget.24834)
Supplement: Supplementary file 1 [file oncotarget-09-17980-s001.pdf]

Drosophila PEBP1 inhibits intestinal stem cell aging via suppression of ERK pathway

SUPPLEMENTARY MATERIALS

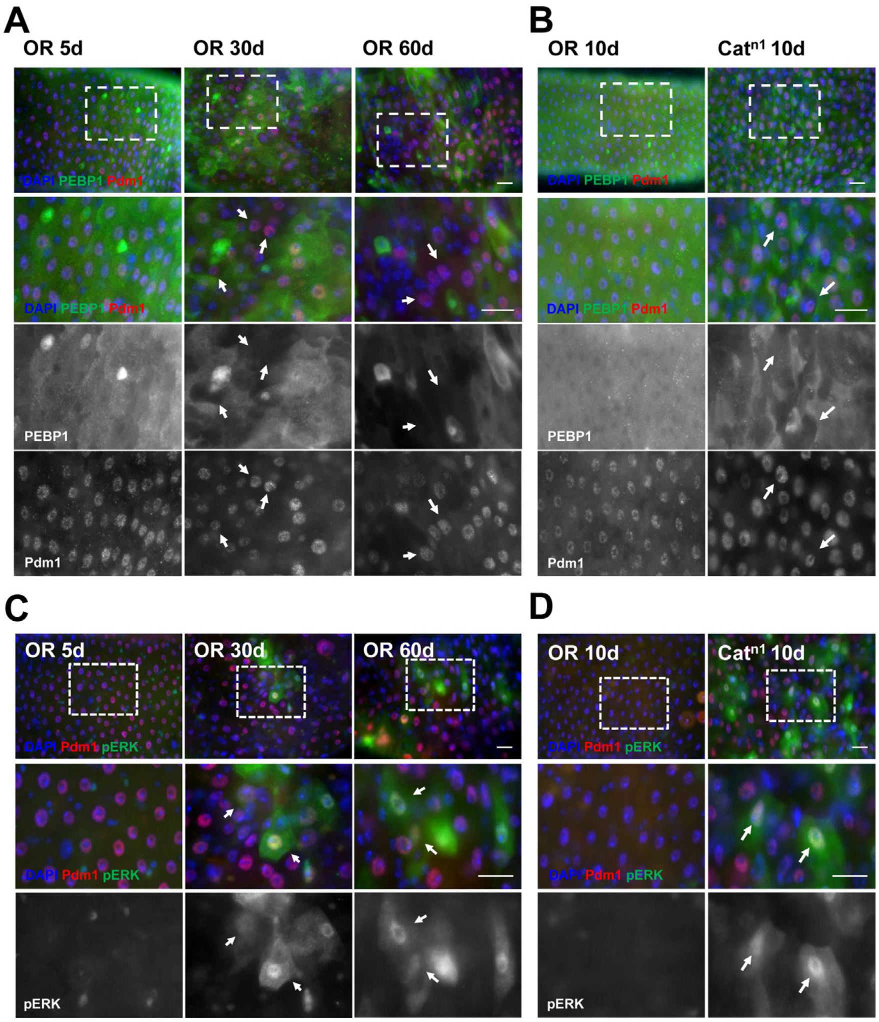

Supplementary Figure 1: Age-related changes of PEBP1 expression and ERK activity in ECs of aged and oxidative stressed midguts. (**A**) PEBP1 expression decreased in ECs of 30- and 60-day-old flies as compared with 5-day-old flies. Guts of OR flies were stained with anti-PEBP1 (green), anti-Pdm1 (red), and DAPI (blue). The lower images are the enlarged images of the white dashed rectangle in the upper panel. White arrows indicate ECs showing decreased PEBP1 (decreased PEBP1 and Pdm1^{+} cells). d, day. Scale bar, 20 µm. (**B**) The PEBP1 expression decreased in ECs of catalase mutant flies (*Cat^{n1}*/+) guts. Guts of 10-day-old OR or *Cat^{n1}*/+ flies were stained with anti-PEBP1 (green), anti-Pdm1 (red), and DAPI (blue). The lower images are the enlarged images of the white dashed rectangle in the upper panel. Arrows indicate ECs showing decreased PEBP1 (decreased PEBP1 and Pdm1^{+} cells). d, day. Scale bar, 20 µm. (**C**) Increased pERK expression was detected in ECs of 30- and 60-day-old flies as compared with 5-day-old flies. Guts of OR flies were stained with anti-pERK (green), anti-Pdm1 (red), and DAPI (blue). The two lower panels are enlarged images of the white dashed rectangle in the upper panel. White arrows indicate ECs showing increased ERK activity (pERK^{+} and Pdm1^{+}). d, day. Scale bar, 20 µm. (**D**) The ERK activity increased in ECs of catalase mutant flies (*Cat^{n1}*/+) guts. Guts of 10-day-old OR or *Cat^{n1}*/+ flies were stained with anti-pERK (green), anti-Pdm1 (red), and DAPI (blue). The two lower panels are enlarged images of the white dashed rectangle in the upper panel. White arrows indicate ECs showing increased ERK activity (pERK^{+} and Pdm1^{+}). d, day. Scale bar, 20 µm.

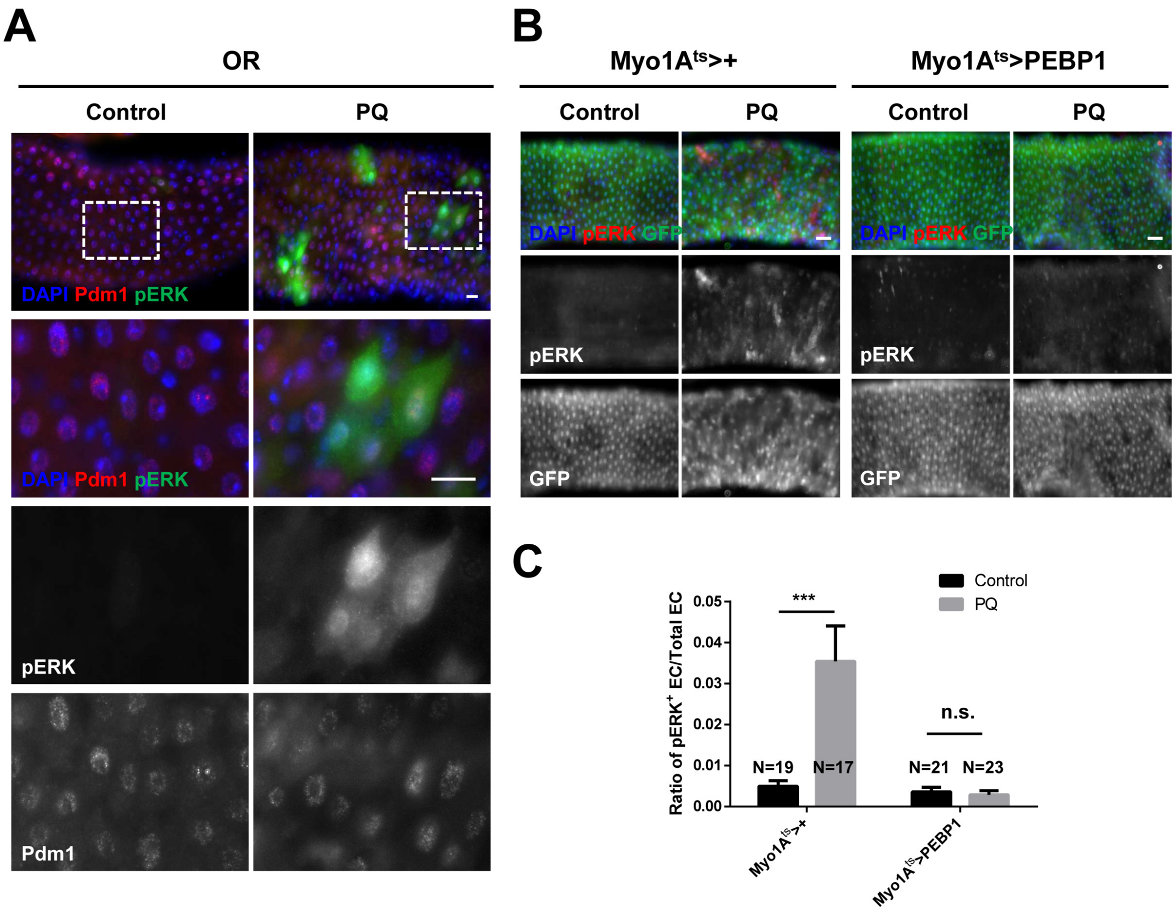

Supplementary Figure 2: EC-specific overexpression of PEBP1 reduces ERK activity under oxidative stress condition. (**A**) The number of $pERK^+$ ECs was increased by PQ treatment. After 10 mM PQ treatment, guts were dissected and stained with anti-pERK (green), anti-Pdm1 (red), and DAPI (blue). The three bottom panels are enlarged images of the white dashed rectangle in the upper panel. Scale bar, 10 μm. Suc, Sucrose. PQ, paraquat. (**B**) The number of $pERK^+$ ECs increased by PQ was reduced by EC-specific PEBP1 overexpression. After induction for 5 days at 29° C, flies were fed 10 mM PQ for 18 h. After 10 mM PQ treatment, guts were dissected and stained with anti-GFP (green), anti-pERK (red), and DAPI (blue). Scale bar, 20 μm. Suc, Sucrose. PQ, paraquat. (**C**) The ratio of $pERK^+$ ECs/Total ECs in EC-specific PEBP1 overexpressed guts (*$Myo1A^{ts}$>PEBP1*) was reduced after PQ treatment as compared to control (*$Myo1A^{ts}$*>+). The number of ECs with pERK expression ($pERK^+$ and GFP^+ cells) was counted under 400× magnification. Data (mean ± SEM) collated from 17-23 guts. N, number of counted guts. *P* values were calculated using a Student's *t*-test. $^{***}p < 0.001$, n.s. not significant.

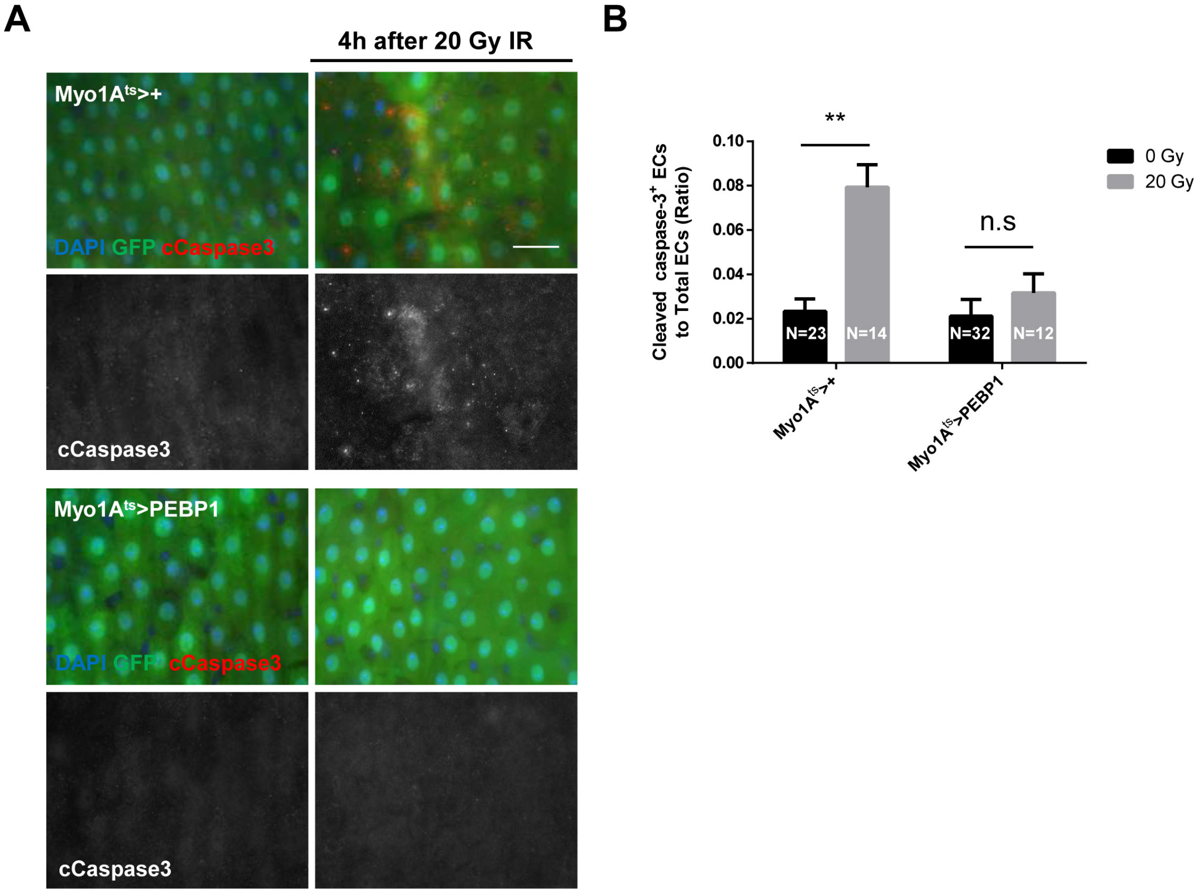

Supplementary Figure 3: EC-specific overexpression of PEBP1 reduces EC death after radiation exposure. (**A**) Radiation-induced EC death was reduced by EC-specific PEBP1 overexpression. After exposure to 20 Gy radiation, the cleaved caspase-3^{+} ECs increased in control guts (*Myo1A^{ts}>+*) but decreased in EC-specific PEBP1 overexpressed guts (*Myo1A^{ts}>PEBP1*). After induction for 2 days at 29° C, flies were irradiated using γ-irradiation machine. At 4 h after radiation exposure, guts of *Myo1A^{ts}>+* or *Myo1A^{ts}>PEBP1* were dissected and stained with anti-GFP (green), anti-cleaved caspase-3 (red), and DAPI (blue). IR, Irradiation. Scale bar, 20 μm. (**B**) The ratio of cleaved caspase-3^{+} ECs/total ECs in EC-specific PEBP1 overexpressed guts was reduced after exposure to 20 Gy radiation for 4 h as compared to control. The number of ECs with cleaved caspase-3 expression was counted under 400× magnification. Data (mean ± SEM) collated from 12–32 guts. N, number of counted guts. *P* values were calculated using a Student's *t*-test. $^{**}p < 0.01$, n.s. not significant.

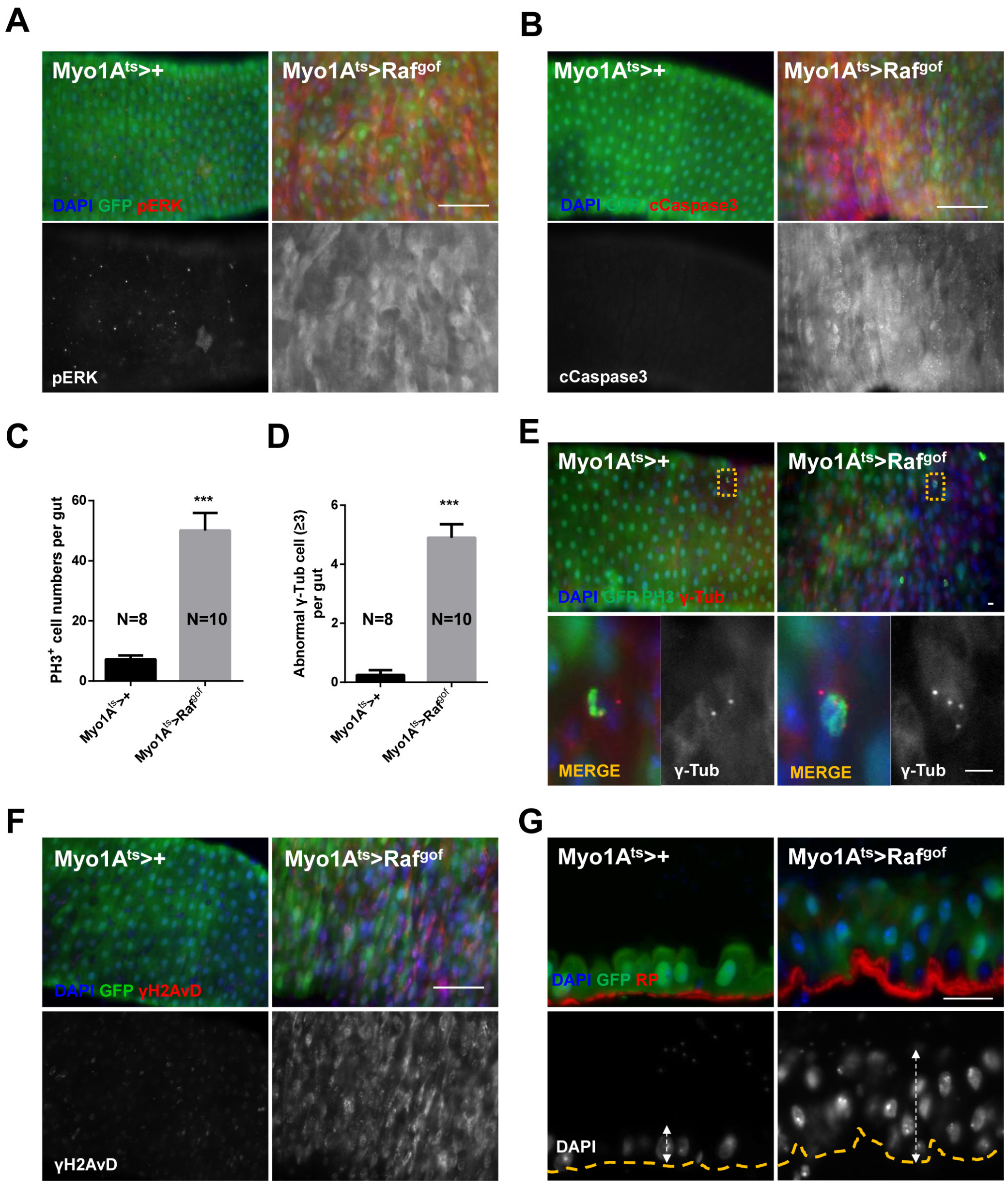

Supplementary Figure 4: Activated Raf overexpression in ECs causes ERK activation, EC death, and age-related phenotypes of ISCs. (**A**) Overexpression of activated Raf (Raf^{gof}) results in the increased activity of ERK in ECs. After induction for 7 days at 29° C, guts of $Myo1A^{ts}>+$ or $Myo1A^{ts}>Raf^{gof}$ were stained with anti-GFP (green), anti-pERK (red), and DAPI (blue). Scale bar, 50 μm. (**B**) The expression of cleaved caspase-3 increased in EC-specific Raf^{gof} overexpressed guts. After induction for 7 days at 29° C, guts of $Myo1A^{ts}>+$ or $Myo1A^{ts}>Raf^{gof}$ were stained with anti-GFP (green), anti-cleaved caspase-3 (red), and DAPI (blue). Scale bar, 50 μm. (**C**) The number of mitotic ISCs increased in EC-specific Raf^{gof} overexpression. After induction for 7 days at 29° C, guts of $Myo1A^{ts}>+$ or $Myo1A^{ts}>Raf^{gof}$ were stained with anti-GFP (green), anti-PH3 (red) and DAPI (blue). The number of $PH3^{+}$ cells was counted in whole guts. Data (mean ± SEM) collated from 8–10 guts. N, number of counted guts. *P* values were calculated using a Student's *t*-test. $^{***}p < 0.001$ based on comparison to the control guts. (**D**) The number of mitotic ISCs with supernumerary centrosome (≥3) increased in EC-specific Raf^{gof} overexpressed guts. The number of γ-Tubulin (γ-Tub) signal was counted in mitotic ISCs of guts. Data (mean ± SEM) were collated from 8–10 guts. N, number of counted guts. *P* values were calculated using a Student's *t*-test. $^{***}p < 0.001$ based on comparison to the control guts. (**E**) Images of increased supernumerary centrosome in EC-specific Raf^{gof} overexpressed guts. After induction for 7 days at 29° C, guts of $Myo1A^{ts}>+$ or $Myo1A^{ts}>Raf^{gof}$ were stained with anti-GFP (green), anti-PH3 (green), anti-γ-Tubulin (γ-Tub, red), and DAPI (blue). The two lower panels are enlarged images of the yellow dashed rectangle in the upper panel. Scale bar, 5 μm. (**F**) Gamma-H2AvD accumulation of ISCs increased in EC-specific Raf^{gof} overexpressed guts. After induction for 7 days at 29° C, guts of $Myo1A^{ts}>+$ or $Myo1A^{ts}>Raf^{gof}$ were stained with anti-GFP (green), anti-γH2AvD (red), and DAPI (blue). Scale bar, 50 μm. (**G**) Sagittal view of midgut epithelium hyperplasia induced by EC-specific Raf^{gof} overexpression. After induction for 7 days at 29° C, guts of $Myo1A^{ts}>+$ or $Myo1A^{ts}>Raf^{gof}$ were stained with anti-GFP (green), RP (red) and DAPI (blue). Yellow lines indicate RP of upper panels and white arrows indicate thickness. Original magnification is 400×. Scale bar, 20 μm.

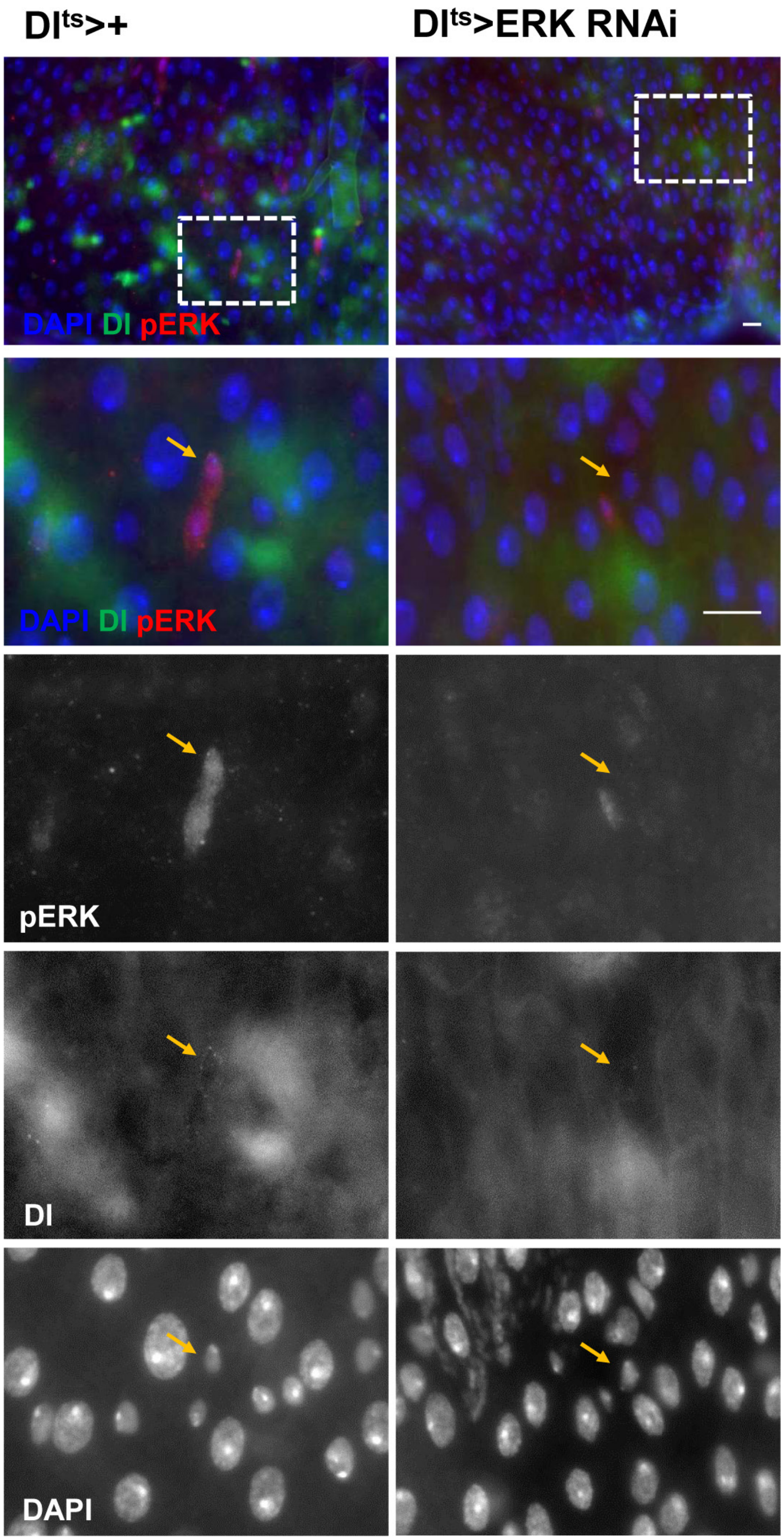

Supplementary Figure 5: RNAi-mediated down-regulation of ERK is sufficient to reduce ERK signaling activity. ERK activity was decreased in ISCs (Dl^+ cells, yellow arrows of right panels) upon ISC-specific knockdown of ERK using *Dl-Gal80*^{ts}. After induction for 7 days at 29° C, guts of *Dl*^{ts}>+ or *Dl*^{ts}*>ERK RNAi* were stained with anti-Dl (green), anti-pERK (red), and DAPI (blue). Yellow arrows indicate ISCs (Dl^+ cells). The four lower panels are enlarged images of the white dashed rectangle in the upper panel. Scale bar, 10 μm.

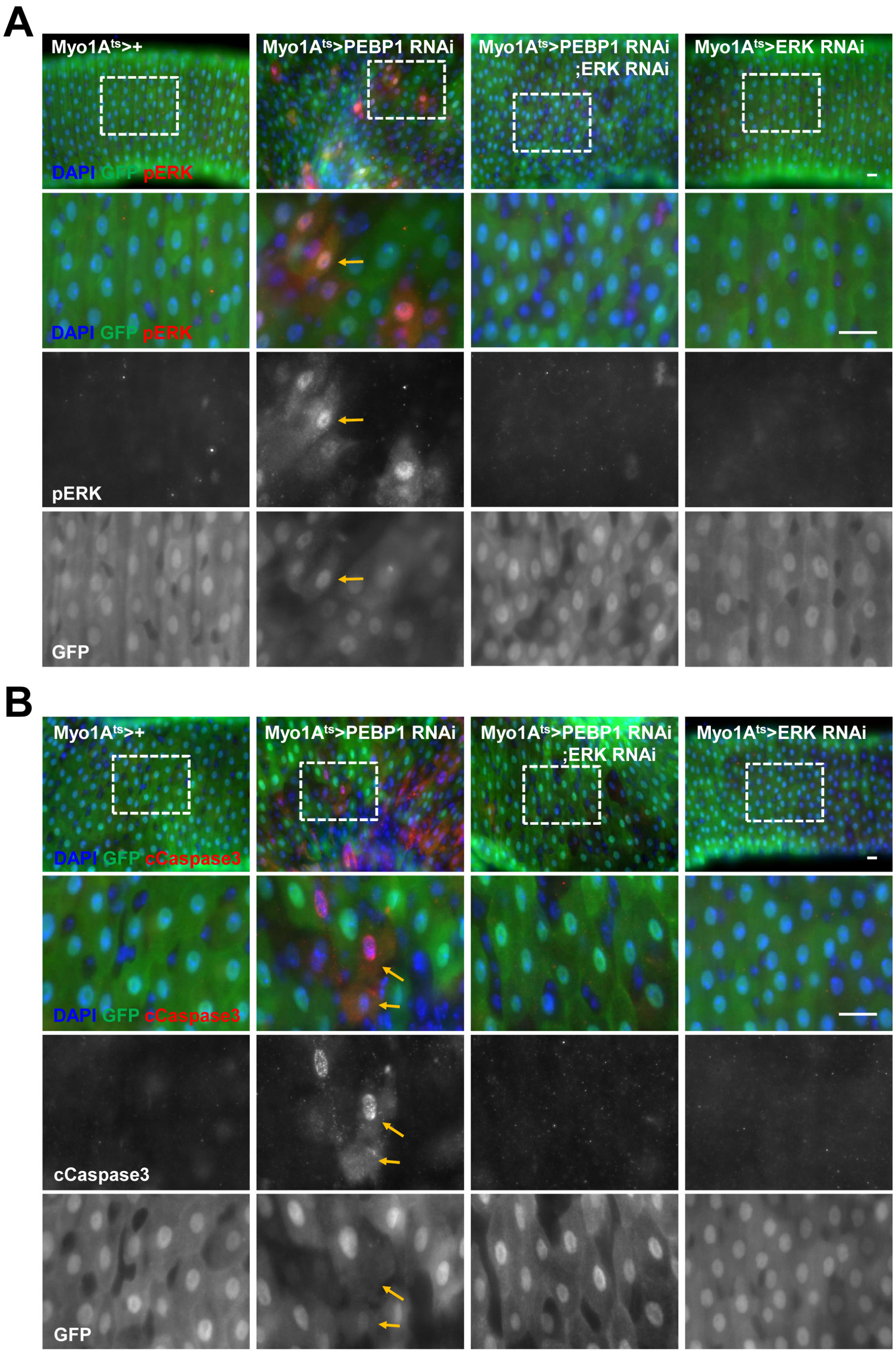

Supplementary Figure 6: Inhibition of ERK activity rescues the phenotypes induced by PEBP1 depletion: expression of pERK and cleaved caspase-3. (**A**) Magnified images of Figure 5A. After induction for 7 days at 29° C, guts were stained with anti-GFP (green), anti-pERK (red) and DAPI (blue). The three lower panels are enlarged images of the white dashed rectangle in the upper panel. Yellow arrow indicates pERK^{+} EC (pERK^{+} GFP^{+} cell). Scale bar, 10 µm. (**B**) Magnified images of Figure 5B. After induction for 7 days at 29° C, guts were stained with anti-GFP (green), anti-cleaved caspase-3 (red) and DAPI (blue). The three lower panels are enlarged images of the white dashed rectangle in the upper panel. Yellow arrows indicate cleaved caspase-3^{+} EC (cleaved caspase-3^{+} GFP^{+} cell). Scale bar, 10 µm.

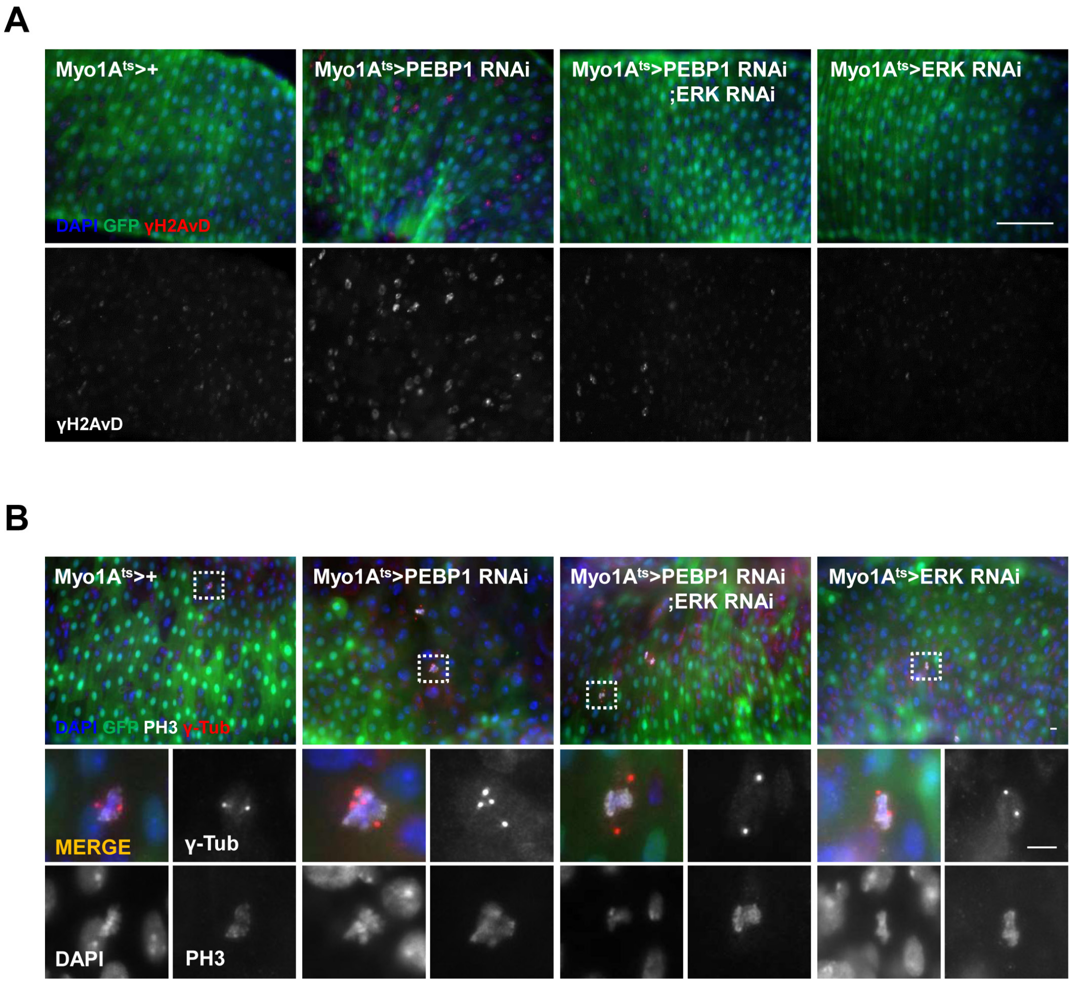

Supplementary Figure 7: Inhibition of ERK activity rescues the phenotypes induced by PEBP1 depletion: DNA damage accumulation and centrosome amplification. (**A**) The increase in γH2AvD accumulation of EC-specific PEBP1 knockdown guts was compensated by EC-specific ERK knockdown. After induction for 7 days at 29° C, guts were stained with anti-GFP (green), anti-γH2AvD (red) and DAPI (blue). Scale bar, 50 µm. (**B**) Representative images of Figure 5D. After induction for 7 days at 29° C, guts were stained with anti-GFP (green), anti-PH3 (white), anti-γ-Tubulin (red) and DAPI (blue). The four lower panels are enlarged images of the white dashed rectangle in the upper panel. Scale bar, 5 µm.
